# Application of Intelligent Response Fluorescent Probe in Breast Cancer

**DOI:** 10.3390/molecules29184294

**Published:** 2024-09-10

**Authors:** Anqi Sheng, Hao Zhang, Qing Li, Shu Chen, Qingshuang Wang

**Affiliations:** 1 College of Life Science and Technology, Changchun University of Science and Technology, Changchun 130013, China; saq1988@126.com (A.S.); zhanghao@cust.edu.cn (H.Z.); 2 Technology Innovation Institute of Jilin Province, Changchun 130012, China; 13944832718@163.com (Q.L.); chenshu7381@163.com (S.C.)

**Keywords:** synthesis, fluorescent probes, application, breast cancer

## Abstract

As one of the leading cancers threatening women’s lives and health, breast cancer is challenging to treat and often irreversible in advanced cases, highlighting the critical importance of early detection and intervention. In recent years, fluorescent probe technology, a revolutionary in vivo imaging tool, has gained attention in medical research for its ability to improve tumor visualization significantly. This review focuses on recent advances in intelligent, responsive fluorescent probes, particularly in the field of breast cancer, which are divided into five categories, near-infrared responsive, fluorescein-labeled, pH-responsive, redox-dependent, and enzyme-triggered fluorescent probes, each of which has a different value for application based on its unique biological response mechanism. In addition, this review also covers the strategy of combining fluorescent probes with various anti-tumor drugs, aiming to reveal the possibility of synergistic effects between the two in breast cancer treatment and provide a solid theoretical platform for the clinical translation of fluorescent probe technology, which is expected to promote the expansion of cancer treatment technology.

## 1. Introduction

Breast cancer poses a significant threat to women’s health, being the most prevalent form of cancer and one of the leading causes of cancer-related deaths among women [1,2]. It affects women globally, irrespective of age, with its incidence increasing as age advances. Breast cancers are classified based on the expression of estrogen receptors (ERs), progesterone receptors (PRs), and human epithelial growth factor receptor 2 (HER2) into three categories: hormone receptor-positive (ER^+^, PR^+^, HER2^−^), HER2^−^rich (ER^−^, PR^−^, HER2^+^), and triple-negative (ER^−^, PR^−^, HER2^−^) [3,4]. Notably, over 90% of breast cancer diagnoses reveal non-disseminated tumors at the initial clinical evaluation. For patients without evident distant metastases, therapeutic interventions primarily focus on the complete eradication of the malignant growth and the prevention of recurrence. Triple-negative breast cancer demonstrates a markedly higher susceptibility to recurrence compared to other subtypes. This is supported by a 5-year survival rate of 85% for individuals diagnosed with stage I breast cancer, which contrasts significantly with the 94% to 99% survival rates reported for hormone receptor-positive and ERBB2-positive patient cohorts. In the context of metastatic disease, the median survival duration for triple-negative breast cancer is estimated to be approximately one year, which is significantly lower than the roughly five-year survival timelines characteristic of other breast cancer subtypes [5]. Despite advancements in medical technology that have led to notable improvements in the diagnosis, treatment, and prevention of cancer, resulting in enhanced patient survival rates, better prognoses, and a reduction in overall mortality, breast cancer continues to be the fifth leading cause of cancer-related deaths worldwide. It is responsible for more disability-adjusted life years lost than any other cancer, profoundly impacting women’s lives and health. Breast cancer ranks among the three most common cancers globally; however, an early diagnosis and treatment are associated with curative outcomes [6]. Furthermore, the pursuit of precision in breast cancer diagnostics relies heavily on the application of various imaging modalities, which serve as a cornerstone in this field. These modalities collectively provide detailed anatomical and functional information, facilitating accurate localization, characterization, and staging of breast malignancies. This multifaceted approach to imaging ensures a comprehensive understanding of the disease state, which is crucial for developing personalized treatment strategies tailored to individual patient needs [7]. Nonetheless, many of these imaging modalities possess inherent limitations in spatial resolution and targeting capabilities, which can hinder their ability to accurately identify diseased tissue.

In recent years, the application of fluorescent probes as a technique for tumor visualization in the early diagnosis of tumors has been extensively studied [8,9,10]. As fluorescent sensors, these probes utilize fluorescent substances as indicators to detect specific molecules by monitoring changes in fluorescence signals. Typically, fluorescent probes consist of three components: a recognition group, a binding group, and a signal reporter group. These critical components determine the probes’ specificity and selectivity for recognizing the target substance. The signal reporter group is responsible for translating the alterations in the chemical environment, resulting from the interaction between the recognition group and the target substance, into a fluorescent signal. In contrast, the binding group serves to connect the recognition group to the signal reporter group [11,12,13]. Smart-response fluorescent probes are pivotal for tumor visualization and diagnoses. These probes can leverage the characteristics of the tumor microenvironment or utilize nanoparticles to achieve visualization, making them a prominent area of research in recent years.

Fluorescent nanoprobes, as a cutting-edge light-sensitive nanotechnology product, have demonstrated significant potential across various biomedical fields, including small-molecule recognition, biomolecule tracking, intracellular structure visualization, and in vivo disease diagnoses. This positions them as an ideal alternative to traditional organic fluorescent dyes. This review comprehensively evaluates the precise design and synthesis of various functional fluorescent nanoprobes, with a particular focus on their application in the early diagnosis of breast cancer, a highly prevalent malignant tumor among women. The aim is to establish a robust theoretical framework and scientific basis for advancing fluorescent nanoprobe technology into clinical practice, thereby facilitating its widespread application and technological innovation in the realm of precision medicine.

## 2. Use of Different Functionalized Fluorescent Probes in Breast Cancer

In recent years, researchers have modified fluorescent probes to enhance their responsiveness to the varying microenvironments of tumors. This advancement allows for the more accurate and rapid identification of tumor sites, facilitating early diagnoses of breast cancer in clinical settings.

### 2.1. Synthesis of Near-Infrared Responsive Fluorescent Probes and Their Application in Breast Cancer

Fluorescence imaging alone absorbs light in the visible tissue region when detecting tumor sites, resulting in attenuation, scattering, and a reduction in the light signal. This leads to defects in imaging with fluorescent probes [14]. Near-infrared (NIR) fluorescent probes effectively address these shortcomings by increasing the depth of fluorescence imaging and reducing the interference associated with tissue-induced absorption, scattering, and autofluorescence, thereby improving the accuracy of fluorescence imaging [15]. Currently, NIR-responsive fluorescent probes have been extensively studied and are expected to be utilized in clinical applications for the real-time diagnosis of tumors. They aim to provide more accurate evidence for tumor treatment, ultimately improving the survival rate of patients with tumors [16]. Based on the emission wavelength, NIR fluorescence imaging can be divided into two regions: the near-infrared one region (NIR-I, 700–900 nm) and the near-infrared two region (NIR-II, 1000–1700 nm) [17].

Wu et al. [18] synthesized a novel fluorescent probe utilizing methionine (Met) and indocyanine green (IGG) derivatives. IGG is an FDA-approved tricarbocyanine dye characterized by its infrared-absorbing properties, functioning as a near-infrared (NIR) fluorescent dye within the wavelength range of 700–900 nm. The NIR fluorescent dye was produced through the aminocarbamylation of one of the IGG derivatives, resulting in aminocarbamylated IR-782 (NH_2_-IR-782). This NH_2_-IR-782 was then introduced into a dimethyl sulfoxide (DMSO) solution containing 1-ethyl-3-(3-dimethylaminopropyl) carbodiimide hydrochloride (EDC) and N-hydroxysuccinimide (NHS) to activate the amino group in NH_2_-IR-782, facilitating the incorporation of Met into the NH_2_-IR-782 structure to yield the final product, Met-IR-782. Their findings demonstrated that Met-IR-782 exhibits excellent fluorescence stability, good photothermal conversion efficiency under 780 nm laser irradiation, and specific targeting to the human breast cancer cell line MCF-7, effectively inhibiting the proliferation of MCF-7 cells through the photothermal effect.

**Figure 1:**
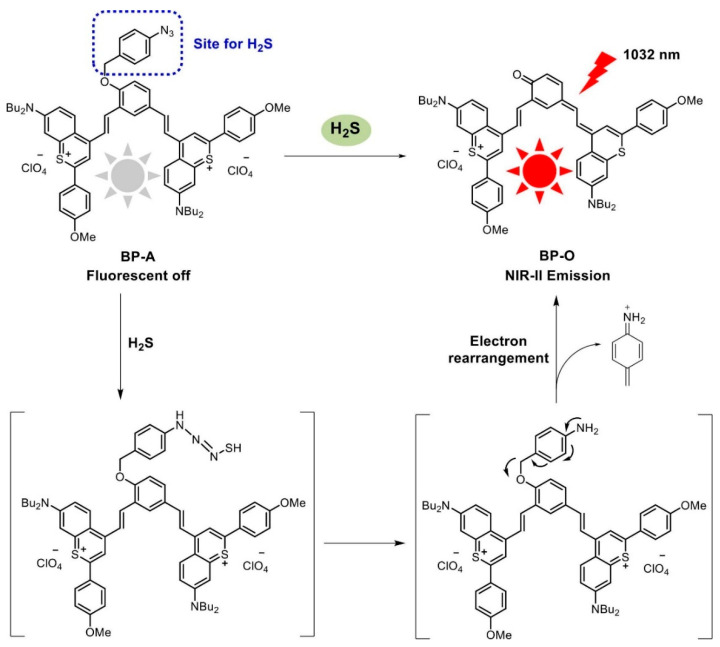
Structural synthesis of the NIR-II fluorescent probe BP-A and the mechanism of response to H_2_S [24]. Copyright 2023, ELSEVIER.

**Figure 2:**
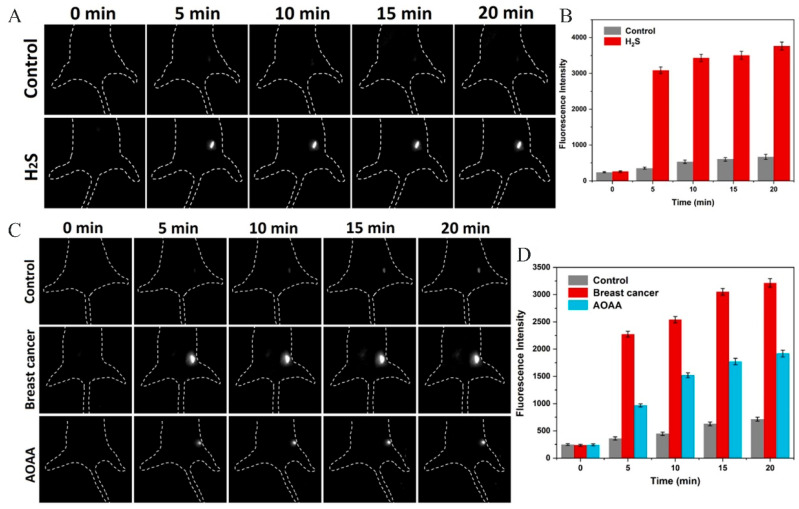
(**A**) Fluorescence imaging of exogenous H_2_S in non-homozygous mice. (**B**) Relative fluorescence intensity. (**C**) Fluorescence imaging of exogenous H_2_S in hormonal mice. (**D**) Relative fluorescence intensity [24]. Copyright 2023, ELSEVIER.

Compared to NIR-I, NIR-II is more widely utilized in breast cancer detection due to its relatively low absorption, scattering, and autofluorescence in biological tissues, which allows for significant penetration depth and facilitates deep tissue imaging [19]. In contrast to conventional NIR-II fluorescent imaging materials, such as quantum dots, carbon nanotubes, and metal nanoparticles, organic fluorescent probes are promising candidates for fluorescence imaging because of their low cytotoxicity, robust photostability, and favorable biocompatibility [20]. Zhou et al. [21] developed a fluorescent matrix based on chalcone, which was designed and synthesized to exhibit specific anti-tumor activities for both in vivo and in vitro diagnoses and treatment of breast cancer. The efficacy of this fluorescent probe is attributed to the precise quantification of hydrogen sulfide (H_2_S) in vivo using the chalcone fluorescent matrices. The intracellular levels of H_2_S are closely associated with the proliferation and apoptosis of breast cancer cells. H_2_S, recognized as an essential endogenous signaling gas and redox molecule, possesses a wide array of physiological and protective properties in biological systems [22]. A variety of disease phenotypes may be associated with abnormal endogenous H_2_S levels; therefore, the sensitive and quantitative detection of H_2_S levels is essential for accurate diagnoses and prognostic assessment of breast cancer [21,23]. Similarly, Yang et al. [24] developed a near-infrared II (NIR-II) fluorescent probe, BP-A, to accurately detect intracellular H_2_S levels in breast cancer cells and to investigate its role in breast cancer progression. The response mechanism of the NIR-II fluorescent probe, BP-A, is fundamentally non-fluorescent due to the quenching effect of the azide moiety. The reduction of the azide group by H_2_S, followed by the rapid cleavage of the imine intermediate, restores the NIR-II fluorescence of BP-O. The significant Stokes shift of the BP-A probe alleviates the issue of self-absorption and minimizes interference from autofluorescence (Figure 1). Additionally, BP-A exhibited a significant Stokes shift, demonstrated prompt responsiveness, and showcased prominent NIR-II emission at 1032 nm. These attributes enabled the probe to quantitatively assess hydrogen sulfide (H_2_S), both introduced and naturally occurring, with high sensitivity and specificity in live murine subjects. Notably, BP-A exhibited intense NIR-II fluorescence in tumor-bearing mice, while healthy mice did not display any such signal. This finding highlights its superior capability to differentiate between non-diseased and breast cancer-affected mice, as illustrated in Figure 2. Collectively, these discoveries emphasize the potential of BP-A as a promising agent for the non-invasive monitoring of H_2_S levels and its applicability in the differential diagnosis of breast cancer through high-resolution NIR-II imaging techniques. In addition, dyes, organic semiconductor polymers, rare earth elements, and anthocyanine-based NIR fluorescent probes have been used to detect breast cancer (Table 1).

In conclusion, the in vivo processing and application of near-infrared fluorescent materials must thoroughly consider their biocompatibility and fluorescence quantum efficiency and the tunability of both excitation and emission wavelengths within the near-infrared region. It is essential to select materials with varying properties based on the specific tumor types for effective visual tumor imaging.

### 2.2. Synthesis of pH-Responsive Fluorescent Probes and Their Application in Breast Cancer

Dysregulated pH has been recognized as a universal hallmark of the tumor microenvironment, which can facilitate dormant metastasis by enhancing cancer cell migration [29]. pH-Responsive probes leverage this low pH to improve metastasis imaging while reducing background fluorescence [30]. Numerous innovative pH-responsive probes have been developed for tumor imaging [31,32]. The fluorescent moieties that constitute the majority of pH-responsive fluorescent probes are now broadly categorized into the following groups: rhodamine analogs [33,34], coumarins [35,36], benzoxazoles [37], and naphthylimides [38], as well as pH-sensitive dye 1 [39]. For further details, please refer to Table 2.

Wu et al. [33] utilized 3-aminophenyl boronic acid-modified gold@rhodamine B nanoparticles to achieve the specific recognition of salivary acids in breast cancer cells, successfully imaging MCF-7 human breast cancer cells. Rhodamine B serves as a crucial fluorescent dye due to its high intensity and stability. Additionally, Cao et al. [35] developed an innovative pH-responsive fluorescence-enhanced nanogel named CDDPHA-CD@AUR, specifically designed for precision imaging and cell-targeted therapy of breast cancer. This nanogel employs a dual targeting strategy: firstly, it utilizes the specific recognition capabilities of the hyaluronic acid (HA) receptor for active targeting; secondly, the affinity of orange peel oleoresin (AUR) enhances binding to breast tumor cells, significantly improving the uptake efficiency of CDDPHA-CD@AUR by breast cancer cells. The fluorescent properties of AUR enable the nanogel to be visualized and tracked at the cellular level, facilitating the observation of its intracellular localization and distribution. In its construction, cisplatin (CDDP) not only acts as a cross-linking agent to stabilize the entire nanostructure but also serves as a therapeutic agent, ensuring the stability of the nanogel by chelating with the carboxyl group of HA. Most importantly, this cross-linking mechanism allows for the regulation of CDDP release in response to pH changes within the tumor microenvironment, enabling intelligent drug delivery. The CDDPHA-CD@AUR was evaluated through in vitro experiments to study its effects on MCF-7 and HK-2 cells and to assess its tumor-suppressive effects and systemic toxicity in hormone-treated mice.

Yu et al. [37] have ingeniously designed and synthesized a novel class of pH fluorescent probes, consisting of 2-piperazine-1-formyl-1,3-benzothiazole (PBZ) linked by 7-nitro-1,2,3-benzoxadiazole (NBD), referred to as NBD-pbz. In acidic environments (low pH), NBD-pbz undergoes protonation to form NBD-pbz^+^H^+^, which significantly enhances fluorescence at 540 nm, resulting in bright yellow emission. NBD-pbz has been demonstrated to effectively and precisely monitor pH changes within the range of 3.2 to 7.6 through observable color changes and fluorescence signals. Furthermore, NBD-pbz exhibits high reversibility, stability, and biocompatibility, making it an excellent candidate for pH sensing.

Copper ions (Cu^2+^) are essential trace elements, second only to iron and zinc in the human body [40]. However, the excessive accumulation of Cu^2+^ can lead to cell death [41]. Therefore, Zhang et al. [38] synthesized a novel fluorescent probe, NapL, which is based on a 1,8-naphthalene fluorophore as the core and incorporates the Nile Blue (NB) structure through the hydroxyl group at the 4-position and the nitroso group at the 3-position. NapL employs ligand binding to achieve the selective recognition of Cu^2+^ while simultaneously modifying the pH using the imide skeleton present in the NB structure. Based on this, NapL was utilized in breast cancer MCF-7 cells, where Cu^2+^ levels were determined under neutral conditions.

Recently, fluorescence-based pH sensing materials have garnered significant attention due to their high sensitivity, excellent biocompatibility, pH reversibility, compact size, and affordability [42]. However, this technology does have limitations, including susceptibility to interference from dehydroascorbic acid, ascorbic acid, and methylglyoxal (DHA, AA, MGO) [43,44]. The fluorescence output is particularly sensitive near neutral pH, which affects measurement accuracy and limits the capability for instantaneous monitoring. Additionally, the relatively long response time, typically exceeding five minutes, may pose challenges in acidic environments with a pH below approximately 5.5 [42].

### 2.3. Synthesis of Redox Fluorescent Probes and Their Application in Breast Cancer

**Figure 3:**
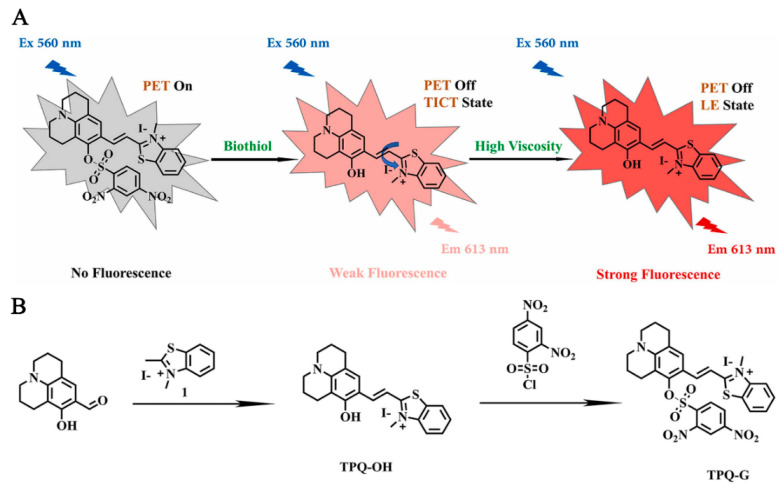
Sensing mechanism of TPQ-G (**A**) and its synthesis (**B**) [47]. Copyright 2022, ELSEVIER.

Redox fluorescent probes play a crucial role in various medical fields, particularly in the treatment of breast cancer [39]. Currently, a prevalent approach to breast cancer therapy involves loading anti-cancer drugs into drug carriers, which are typically composed of nanoparticles. These nanoparticles facilitate the targeted delivery of anti-cancer drugs to cancer cells during treatment, necessitating the real-time detection and monitoring of drug release and efficacy. This approach not only enhances the effectiveness and precision of treatment but also reduces the toxicity and side effects associated with the drugs for patients [45,46]. Zhao et al. [47] developed a fluorescent probe, TPQ-G, which can monitor the levels of GSH in breast cancer cells. TPQ-G reacts with the elevated GSH levels present in tumors, resulting in increased fluorescence intensity. Consequently, this enhancement improves the effectiveness of imaging (Figure 3).

**Figure 4:**
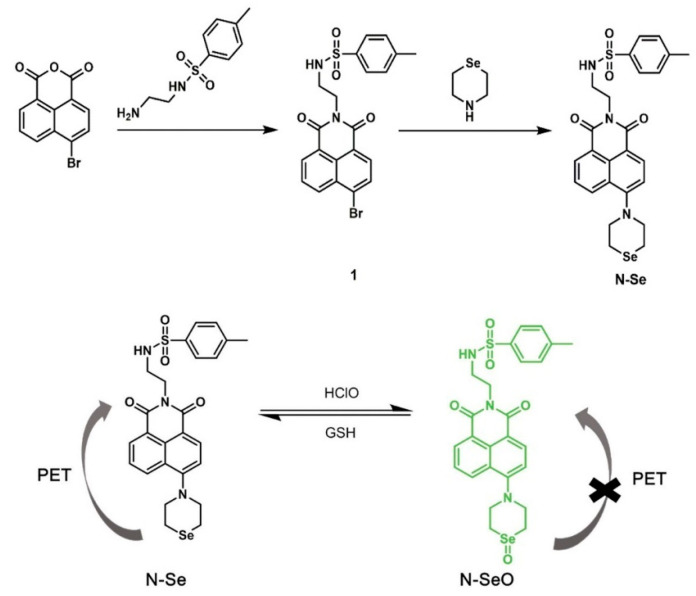
Synthesis of endoplasmic reticulum-targeted fluorescent probe N-Se and its redox reaction process [48].

Dihydroacetylacetonate-based fluorescent probes, which are commonly utilized as redox fluorescent probes, are frequently employed to monitor the redox status of breast cancer cells due to their rapid reduction, resulting in fluorescence enhancement. These probes are typically synthesized by combining dihydroacetylacetone molecules with fluorescent moieties through chemical methods such as nucleophilic substitution and coupling reactions. The endoplasmic reticulum-targeted fluorescent probe developed by Tang et al. [48] is capable of undergoing multiple reversible redox cycles under the regulation of ClO^−^ and GSH, successfully detecting exogenous ClO^−^ and monitoring dynamic changes in the redox state of the ER in real-time (Figure 4).

Redox fluorescent probes of the imidazole–phosphorus–copper class can interact with intracellular redox molecules, thereby altering their fluorescence properties. This characteristic can be utilized to evaluate the efficacy of breast cancer drugs. The synthesis of such probes typically involves the preparation of compounds containing imidazole and phosphorus groups, followed by the introduction of copper ions, which usually entails nucleophilic substitution and coupling reactions. Similarly, sulfhydryl probes can specifically bind to sulfhydryl compounds present in cancer cells, facilitating the detection of oxidative stress and the assessment of anti-cancer drugs. The synthesis of sulfhydryl-based probes necessitates the design of a sulfhydryl-containing compound and the selection of a target molecule for the reaction, which also generally involves nucleophilic substitution and coupling reactions [49]. All these fluorescent probes can be employed for tumor cell imaging, monitoring drug delivery, studying the tumor microenvironment, and evaluating the therapeutic effects of anti-cancer drugs, thereby demonstrating significant medical value in breast cancer treatment. Additionally, the synthesis of various other redox probes and their applications in breast cancer are discussed in Table 3.

### 2.4. Synthesis of Enzyme-Responsive Fluorescent Probes and Their Application in Breast Cancer

Enzymes, as quintessential biocatalysts in nature’s evolutionary orchestration, are indispensable orchestrators of vital physiological processes. Within the complex milieu of biological systems, enzymes are intimately involved in a broad array of biochemical reactions, serving as key mediators of diverse biological phenomena and their pathological manifestations. Consequently, the prospect of monitoring biological occurrences and forecasting diseases through the detection of enzyme activity emerges as a viable investigative strategy. In particular, enzymes have garnered significant interest as potential biomarkers within the oncological landscape. The accurate determination of the spatiotemporal localization and expression levels of these enzymes within live cancer cells is critical for the timely detection of cancer at its inception and for real-time monitoring of therapeutic efficacy [53]. Nonetheless, traditional fluorescent probes exhibit limitations in terms of responsiveness and specificity, leading to inadequate discrimination between tumor and normal tissue profiles and necessitating additional or multiple surgical procedures [54,55]. Enzyme-responsive fluorescent probes, on the other hand, are highly sensitive for detecting low levels of enzymes and are selective for specific enzyme species and activities [56]. Therefore, it is essential to quantify and image cellular enzymes to enhance our understanding of their mechanisms, facilitate drug discovery, advance biological research, and improve clinical diagnoses.

#### 2.4.1. Alkaline Phosphatase Fluorescent Probe

Alkaline phosphatase (ALP), a pivotal enzyme in phosphate metabolism, exhibits broad catalytic activity towards the dephosphorylation of various phosphate esters under alkaline conditions. This enzyme is ubiquitously present in a multitude of mammalian bodily fluids and tissues, highlighting its widespread physiological significance [57]. The physiological function of ALP has long been poorly defined, with some scholars suggesting its association with bone mineralization [58]. However, ALP can be considered an essential biomarker and drug target for numerous human diseases. For instance, abnormal serum ALP levels may serve as a crucial indicator of various conditions, including bone disease, abnormal liver function, breast and prostate cancer, and diabetes mellitus [59,60].

**Figure 5:**
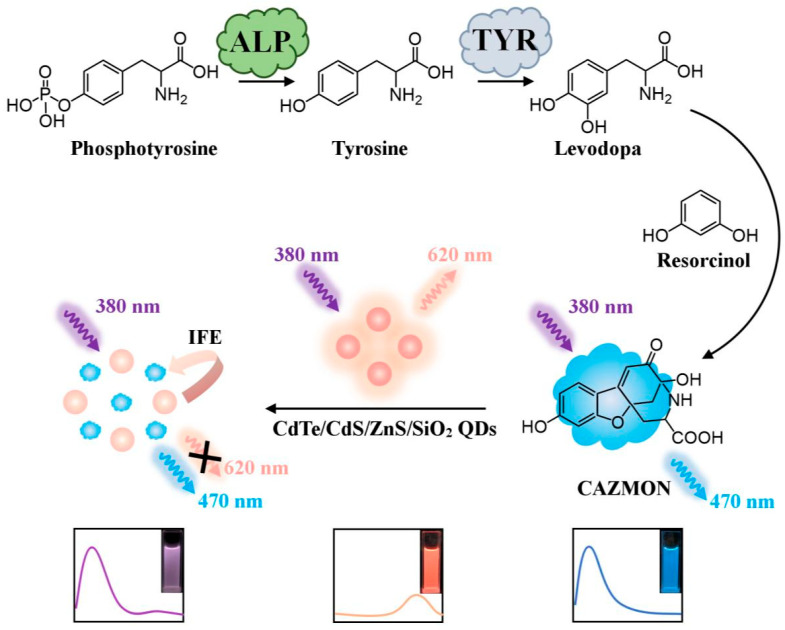
Schematic of enzyme cascade-based ratiometric fluorescent probes for visually monitoring ALP activity. Copyright 2021, ELSEVIER.

Chen et al. [61] proposed a ratiometric fluorescent probe for the visual monitoring of alkaline phosphatase (ALP) activity through an enzymatic cascade reaction involving CdTe/CdS/ZnSO_2_/SiO_2_ quantum dots (QDs) (Figure 5). ALP catalyzes the conversion of phosphotyrosine to tyrosine, which is subsequently catalyzed by tyrosinase (TYR) into levodopa. The reaction product, levodopa, reacts with resorcinol to form carboxylated azaphthalidine (CAZMON), which emits blue fluorescence at 470 nm. Concurrently, CAZMON quenches the red fluorescence emitted by the CdTe/CdS/ZnSO_2_/SiO_2_ QDs at 620 nm. This ratiometric fluorescence change serves as a signal output for quantitative ALP detection, transitioning from red to violet and finally to blue, thus enabling the semi-quantitative detection of ALP. Notably, the probe is capable of directly detecting ALP in human serum and screening for inhibitors without any pretreatment. Consequently, the developed method demonstrates excellent potential for the sensitive and straightforward detection of ALP activity in clinical diagnostics, paving the way for convenient on-site enzyme detection.

#### 2.4.2. Histonease

Cysteine proteases, a class of lysosomal enzymes characterized by significant evolutionary conservation, play a crucial role in tumor progression. Their secretion contributes to the degradation of the extracellular matrix and the disruption of intercellular adhesion molecules, thereby enhancing the invasive capabilities and metastatic spread of tumor cells. In particular, cysteine proteases designated as B, L, C, and S exhibit elevated expression levels in breast cancer tissues, which correlates ominously with poorer patient prognoses [62]. It is important to note that these tissue proteases are not exclusive to cancer cells; they are also produced by immune cells, including macrophages [63]. This information supports the rationale for utilizing molecular imaging agents to target cysteine proteases, thereby improving the accuracy of intraoperative tumor margin delineation during surgical procedures.

Suurs et al. [64] developed a new fluorescence quenching activity probe (qABP), VGT-309, by replacing the Cy5 fluorescent moiety in BMV109 with indocyanine green (ICG) and substituting the sulfo-QSY21 quencher with QC-1. When combined with optical fluorescence imaging, VGT-309 can guide the surgical resection of tumors in homozygous mouse models of breast cancer. Unlike conventional imaging techniques that rely on morphological and structural changes to distinguish tumors, tumor-targeted optical molecular imaging enables real-time in vivo tumor detection based on molecular alterations within the tumors. One hour after the injection of VGT-309, tumors could be detected via fluorescence signals using IVIS spectroscopy. In vivo, the tumor-to-background ratio increased from 6.7 at 1 h to 15.1 at 24 h post-probe injection. This high tumor-to-background ratio also effectively differentiates tumors from normal tissue, facilitating image-guided surgery with two clinical imaging devices as early as 2 h after VGT-309 administration. The flexible imaging window of VGT-309 extends from 2 to at least 24 h after injection, making it an ideal probe for clinical practice. Given that histones are also overexpressed in colorectal, brain, lung, ovarian, and soft tissue sarcomas, VGT-309 may be applicable for tumor delineation and resection across various cancer types. Additionally, fluorescent probes targeting the insulin-like growth factor type 1 receptor, cyclooxygenase-2, acetylcholinesterase, KIAA1363, and carboxylesterase have been utilized in breast cancer (Table 4).

In conclusion, high sensitivity and selectivity are critical requirements for enzyme fluorescent probes, which are essential for the accurate detection of low enzyme levels in early cancer diagnoses. Probes should be designed with appropriate sensing modes that align with the physiological actions and catalytic mechanisms of specific enzymes to enhance the signal-to-noise ratio and improve the sensitivity of novel probes.

## 3. Diagnostic Integration of Fluorescent Probes in Breast Cancer

Breast cancer poses a significant threat to women’s health, with conventional surgery serving as the primary treatment modality, while radiotherapy, chemotherapy, endocrine therapy, and immunotherapy play supportive roles. However, traditional methods often result in side effects, and surgical interventions can negatively impact patients’ quality of life. Additionally, existing breast cancer treatments lack the ability to effectively track and evaluate treatment outcomes. This limitation highlights the urgent need for more targeted and effective drug delivery and evaluation systems in breast cancer therapy. To address these challenges, the concept of “precision medicine” has been proposed, emphasizing “medical visualization, diagnostic and therapeutic integration, low dose, and low toxicity” [70,71]. Consequently, the development of diagnostic-integrated fluorescent probes has garnered significant attention due to their capacity to provide spatiotemporal information regarding drug delivery and, in certain instances, to enable spatiotemporal control in the context of breast cancer treatment [72].

miRNA-21 has been identified as a diagnostic and therapeutic biomarker for breast cancer. Li et al. [73] designed an AuNP-2-OMe DNA probe to detect and inhibit miRNA-21 in breast cancer cells. The results demonstrated that the probe was efficiently introduced into the cells, signaling miRNA-21 as a breast cancer diagnostic marker. Subsequently, the knockdown of miRNA-21 inhibited its function, leading to growth inhibition and apoptotic cell death, thereby facilitating the treatment of breast cancer. In a study conducted by Castilho and colleagues, the potential of a dual-action therapeutic nanoconstruct (DAN) was explored. This construct comprised 21 nm gold nanoparticles conjugated with chlorin e6 (Ce6) and epidermal growth factor (EGF). The binding of Ce6 to the gold nanoparticles ensures stability, while EGF acts as a homing beacon for cancer cells. Utilizing fluorescence spectroscopy and confocal fluorescence microscopy, the research revealed a progressive internalization of these nanoconstructs into cancerous cells at an average rate of approximately 630 billion nanoparticles per minute. Notably, this process did not exhibit any toxic effects on healthy cells. The findings indicate that photodynamic therapy (PDT) facilitated by DAN can effectively induce targeted apoptosis in cancer cells [74]. Rubtsova et al. [75] introduced a novel nanocomposite near-infrared (NIR) fluorescence imaging probe, with the active ingredient being a small molecule, pyropheophorbide a-phosphatidylethanolamine-QSY21, embedded in lipid nanoparticles for in vivo transport and the targeting of phosphatidylcholine-specific phospholipase C overexpression in breast cancer cells to activate the probe. Estrogen (E) and progesterone (Pg) receptors (Rs) have been considered in the design of fluorescent molecular probes with potential therapeutic applications. Barman et al. [76] developed a library of benzothiazole–purine hybridized molecules using quantitative structure–activity relationship (QSAR) technology. Molecular docking was employed to screen for the most promising molecules. Among these, one molecule abbreviated as ‘CPIB’ exhibited blue fluorescence and detected ER-positive cancer cells at a concentration of 1 nM. CPIB induces apoptosis in the same cancer cells at higher concentrations by targeting intracellular microtubules, without affecting normal or ER-negative cells, thereby inducing apoptotic death specifically in the targeted cancer cells.

To mitigate the overheating effect caused by excitation light and enhance the effectiveness of photodynamic therapy (PDT) using up-conversion nanoplatforms, Zeng et al. [77] successfully developed a novel nanoprobe based on up-conversion nanocomposites (T-UCNPs@Ce6@mSiO_2_) that utilizes 808 nm excitation for targeted up-conversion luminescence, magnetic resonance imaging (MRI), and high-efficiency PDT. In this nanocomposite, the photosensitizer (Ce6) is covalently coupled to mesoporous silica, which improves the efficacy of PDT by reducing the fluorescence resonance energy transfer distance and minimizes cytotoxicity by preventing the accidental leakage of Ce6. Compared to UCNPs@mSiO_2_@Ce6, the UCNPs@Ce6@mSiO_2_ configuration significantly enhances the generation of singlet oxygen under 808 nm laser excitation, thereby improving the effectiveness of PDT. The effective development of diagnostic-integrated probes facilitates the loading of various anti-cancer drugs for targeted delivery within the body, significantly increasing drug bioavailability while reducing damage to normal cells. The synthesis and application of fluorescent probes loaded with different types of anti-cancer drugs are summarized in Table 5.

## 4. Summary and Outlook

This research article systematically reviews the preparation methods of various smart fluorescent probes and their innovative applications in the field of breast cancer diagnoses, aiming to establish a solid theoretical foundation for future clinical practice. While the application of smart fluorescent probes in breast cancer detection is primarily limited to laboratory studies, the specific mechanisms by which they inhibit tumor cell growth remain incompletely understood. This indicates that future research should focus on integrating two or more fluorescent probes with distinct smart properties. Such an approach is not only relevant for the accurate identification of breast cancer but also encompasses the evaluation of therapeutic efficacy, while simultaneously exploring the underlying mechanisms of action of these probes on tumor cells. This strategy will not only advance the technology for breast cancer diagnoses and treatment but also provide valuable insights and direction for the development of a new generation of efficient fluorescent probes.

## Figures and Tables

**Table 1:** Different types of near-infrared fluorescent probes in breast cancer.

Fluorescent Probe Type	Probe Molecular Structure	Wavelength	Application	Ref.
Organic Semiconductor Polymers	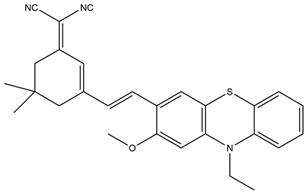	681 nm	Detection of mouse breast cancer cells in homozygous mice for evaluation of the therapeutic effect of cisplatin.	[25]
Rare Earths	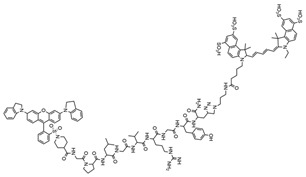	1060 nm and 1340 nm	It can be rapidly eliminated from the hepatobiliary pathway and has been successfully used as a contrast agent for NIR-II in vivo imaging and magnetic resonance imaging (MRI), enabling precise detection of the location of breast cancer lesions.	[26]
Cyanide	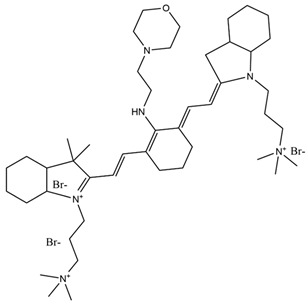	759 nm	Observation of in situ breast tumors and improved surgical accuracy.	[27]
NIR-β-galactosidase (NIR-β-gal-2)	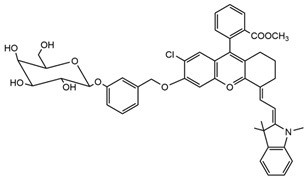	700 nm	Successful removal of an in situ breast tumor by “spraying in situ” and monitoring post-operative recovery.	[28]

**Table 2:** pH-Responsive fluorescent probes with different fluorescent moieties in breast cancer.

Fluorescent Probe Type	Probe Molecular Structure	Application	Ref.
Rhodamine	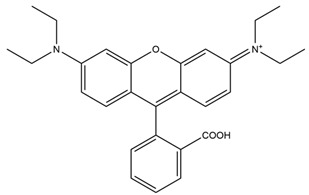	Identification of sialic acid in breast cancer cells and cellular imaging of MCF-7 human cancers.	[33]
Coumarins	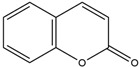	Precision imaging and cell-targeted therapy for breast cancer.	[35]
Benzoxazole	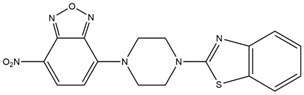	It enhances fluorescence in acidic environments and can be used for cellular imaging.	[37]
Naphthylimides	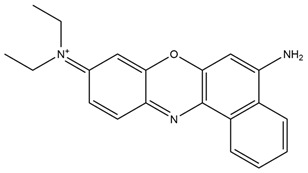	For breast cancer MCF-7 cells and determined Cu^2+^ under neutral conditions.	[38]
pH-Sensitive dye 1	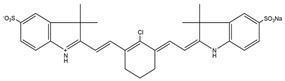	For primary and metastatic breast tumor imaging.	[39]

**Table 3:** Different types of redox probes in breast cancer.

Fluorescent Probe Type	Biomarker	Application	Ref.
Dihydroacetylacetone-based fluorescent probes	DAP-1 and DAP-4	It can be used to monitor levels of ROS in breast cancer cells to help assess the efficacy of anti-cancer drugs.	[50]
Redox fluorescent probes for imidazole–phosphorus–copper analogues	Cu-ImPy and Cu-ImPho	It can be used to monitor levels of ROS in breast cancer cells to help assess the efficacy of anti-cancer drugs.	[51]
Thiol-based probes	AQC, mBBr, and OPA	It can detect GSH and other levels in breast cancer cells, determine drug efficacy, and optimize therapeutic regimens.	[52]

**Table 4:** Application of different enzyme-responsive fluorescent probes in breast cancer.

Fluorescent Probe Type	Probe Molecular Structure	Names of Enzymes and Receptors	Application	Ref.
YQ-L	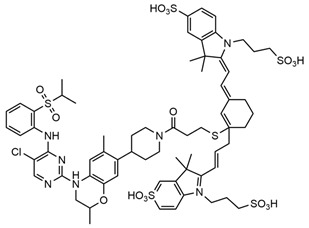	Insulin-like growth factor type 1 receptor (IGF1R)	For the detection of breast cancer.	[65]
Indomethacin	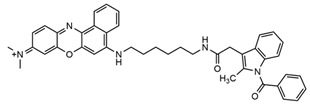	Cyclooxygenase-2 (COX-2)	Tumor imaging applied to mouse models.	[66]
Acetylcholinesterase inhibitors (AchEIs)	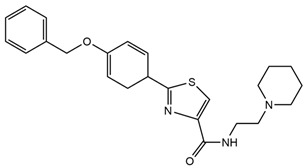	Acetylcholinesterase (AChE), a hydrolyzing enzyme in blood plasma	For identification and inhibition of breast cancer cells.	[67]
Inhibitors AX11890	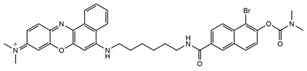	KIAA1363 enzyme	For ultrafast differentiation of breast cancer cells from normal cells in fluorescence imaging with applications to in vivo tissue and tumor imaging.	[68]
CES-Lyso	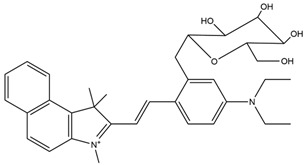	Carboxylesterase (chemistry)	Carboxylesterase activity for differentiating breast cancer cells of different origins and monitoring anti-cancer drug therapy.	[69]

**Table 5:** Fluorescent probes loaded with different types of anti-tumor drugs in breast cancer.

Type of Drugs	Name of Drug	Synthesis and Applications	Ref.
Antibiotics	Adriamycin (DOX)	A microemulsion polymerization in situ loading of Adriamycin (DOX) in iron tetroxide/P (NIPAM-AA-MAPEG) nanogels (MNLs) was designed and prepared with the ability to improve the cell penetration of the drug to achieve controlled release of the drug, to prolong the time of release of the drug, to reduce the toxicity and side effects, and to enable magnetic resonance imaging (MRI), thus enabling the integration of diagnostic and therapeutic treatments.	[78]
		A mSiO_2_-coated AuNBP with a core–shell structure (AuNBP@mSiO_2_) was prepared. The surface was modified with Gd^3+^ chelated with 3-aminopropyl (trimethoxyphenyl) diethylenetriamine tetra-acetic acid (SiDTTA). DOX was subsequently incorporated into the nanoprobe, resulting in MRI-imageable and chemo-photothermally killable breast cancer cells, AuNBP@mSiO_2_-Gd-DTTA@DOX.	[79]
Natural medicine	Salvia divinorum	Inhibition of cytochrome P450 1A1 to enhance breast cancer chemotherapy.	[80]
Antimetabolites	5-FU	A new tumor-targeting nanomedicine (AS1411-T-5-FU) was synthesized using DNA aptamer (AS1411) to modify DNA tetrahedra as a DNA delivery system for loading the anti-tumor drug 5-FU and labelling the DNA tetrahedra with the fluorescent probe Cyanine 5 (Cy5), which enhances the photostability with minimal impact on its basic function, to improve the breast cancer therapeutic efficacy and targeting.	[81]
Natural medicine	Paclitaxel	An integrated nanoplatform for therapeutics and imaging is proposed, which was constructed by co-encapsulation of the photothermal therapeutic agent IR780, the passive targeting drug OA@Fe_3_O_4_, and the chemotherapeutic drug paclitaxel. The nanoparticles exhibited improved photothermal–chemotherapeutic synergistic effects under magnetic targeting guidance, and showed anti-tumor effects in both in vitro and in vivo experiments.	[82]

## Data Availability

Not applicable.
